# Opposing Effects of Semantic Diversity in Lexical and Semantic Relatedness Decisions

**DOI:** 10.1037/a0038995

**Published:** 2015-03-09

**Authors:** Paul Hoffman, Anna M. Woollams

**Affiliations:** 1 Centre for Cognitive Ageing and Cognitive Epidemiology (CCACE), Department of Psychology, University of Edinburgh, and Neuroscience and Aphasia Research Unit (NARU), School of Psychological Sciences, University of Manchester; 2 Neuroscience and Aphasia Research Unit (NARU), School of Psychological Sciences, University of Manchester

**Keywords:** imageability, semantic control, polysemy, context

## Abstract

Semantic ambiguity has often been divided into 2 forms: homonymy, referring to words with 2 unrelated interpretations (e.g., bark), and polysemy, referring to words associated with a number of varying but semantically linked uses (e.g., twist). Typically, polysemous words are thought of as having a fixed number of discrete definitions, or “senses,” with each use of the word corresponding to one of its senses. In this study, we investigated an alternative conception of polysemy, based on the idea that polysemous variation in meaning is a continuous, graded phenomenon that occurs as a function of contextual variation in word usage. We quantified this contextual variation using semantic diversity (SemD), a corpus-based measure of the degree to which a particular word is used in a diverse set of linguistic contexts. In line with other approaches to polysemy, we found a reaction time (RT) advantage for high SemD words in lexical decision, which occurred for words of both high and low imageability. When participants made semantic relatedness decisions to word pairs, however, responses were slower to high SemD pairs, irrespective of whether these were related or unrelated. Again, this result emerged irrespective of the imageability of the word. The latter result diverges from previous findings using homonyms, in which ambiguity effects have only been found for related word pairs. We argue that participants were slower to respond to high SemD words because their high contextual variability resulted in noisy, underspecified semantic representations that were more difficult to compare with one another. We demonstrated this principle in a connectionist computational model that was trained to activate distributed semantic representations from orthographic inputs. Greater variability in the orthography-to-semantic mappings of high SemD words resulted in a lower degree of similarity for related pairs of this type. At the same time, the representations of high SemD unrelated pairs were less distinct from one another. In addition, the model demonstrated more rapid semantic activation for high SemD words, thought to underpin the processing advantage in lexical decision. These results support the view that polysemous variation in word meaning can be conceptualized in terms of graded variation in distributed semantic representations.

Semantic ambiguity and its consequences for language processing has long been an active topic in cognitive science. Traditionally, most research on this topic has focused on the processing of homonyms: words that, by an etymological twist of fate, have come to be associated with two separate and unrelated meanings (e.g., *bark*; Borowsky & Masson, 1996; Kellas, Ferraro, & Simpson, 1988; Rubenstein, Garfield, & Millikan, 1970; Simpson & Burgess, 1985). One long-standing view suggests that each meaning of an ambiguous word is represented as a separate lexical or semantic node in a network, with these nodes competing for activation when the word is processed (Jastrzembski, 1981; Kellas et al., 1988; Morton, 1979; Rubenstein et al., 1970). On this view, processing delays when comprehending homonyms can be understood in terms of competition between their two distinct semantic representations. Homonyms are relatively uncommon, in English at least (accounting for only 7% of words; Rodd, Gaskell, & Marslen-Wilson, 2002). However, another form of ambiguity—polysemy—is much more common. Polysemy refers to more subtle variations in meaning that occur when a particular word is used in different ways in different contexts. For example, the word *chance* can denote a situation governed by luck (“It’s down to chance”), an opportunity that may arise in the future (“I’ll do it when I get a chance”) or a risky option (“Take a chance”). These different “senses” of *chance* are clearly related to one another in meaning, unlike the distinct meanings of *bark*.

How is this type of ambiguity coded in the language system? Some researchers have argued that, just as for homonyms, each sense of a polysemous word has a distinct semantic representation that is unrelated to the word’s other senses (Klein & Murphy, 2001). A limitation of this approach is that it does not take into account the considerable overlap in the meanings of the various senses. Sets of related meanings may be better accommodated by connectionist models of language processing that code semantic knowledge as distributed patterns of activation over a large number of processing units (e.g., Rogers & McClelland, 2004). Within such a framework, related senses can be represented by similar activation patterns. Indeed, a number of connectionist computational models have accounted for effects of polysemy by assuming that the various senses of polysemous words are represented by distinct but overlapping patterns of semantic activation (Armstrong & Plaut, 2008; Kawamoto, 1993; Kawamoto, Farrar, & Kello, 1994; Rodd, Gaskell, & Marslen-Wilson, 2004).

Other researchers have questioned the basic assumption that the variation in the meanings of polysemous words can be segmented neatly into a fixed number of discrete senses (Cruse, 1986; Hoffman, Lambon Ralph, & Rogers, 2013; Kawamoto, 1993; Landauer, 2001). Meanings can vary subtly across contexts and it is often not clear at what point two particular uses of a word become sufficiently distinct to qualify as separate senses or meanings. Rather than attempting to segment variation in meaning into discrete senses, an alternative approach assumes that a word’s meaning can be thought of as varying continuously as a function of the context in which it is used (Hoffman et al., 2013; Landauer & Dumais, 1997). On this view, two uses of the same word are never truly identical in meaning, as their precise connotation in each case depends on the immediate linguistic and environmental context. This approach has two important corollaries. The first is that the semantic system represents the variable meanings of polysemous words in terms of graded variation in a common semantic representation, rather than as distinct nodes in the lexical-semantic network. This continuous view is entirely consistent with distributed approaches to semantic representation, which allow for graded variability in semantic activation when the same word is encountered in different contexts (e.g., McClelland, St. John, & Taraban, 1989). The second is that semantic ambiguity is not considered to be solely a property of a subset of words designated as homonyms or polysemes. Instead, polysemy is viewed as a continuous, graded phenomenon that is present to varying degrees for all words in the language.

Although the continuous approach is entirely consistent with the theoretical stance taken in a number of models of semantic ambiguity (Armstrong & Plaut, 2008; Kawamoto, 1993; Kawamoto et al., 1994; Rodd et al., 2004), the methods used to identify polysemous words in psycholinguistic studies overwhelmingly adhere to the “discrete senses” view. Almost all studies of polysemy have classified words as polysemous based on the number of dictionary definitions they have (e.g., Jastrzembski, 1981; Rodd et al., 2002) or on the number of distinct meanings assigned to them by participants (e.g., Azuma & VanOrden, 1997; Borowsky & Masson, 1996). Recently, Hoffman et al. (2011, 2013) devised an alternative method for quantifying polysemy, based on the idea that variation in a word’s meaning is a continuous function of variation in the contexts in which the word is used. They used latent semantic analysis (Landauer & Dumais, 1997) to quantify this contextual variation. Using this technique, a large text corpus is divided into smaller linguistic contexts and data reduction techniques are used to represent each context as a point in a high-dimensional semantic space, such that the proximity of two contexts indicates their similarity in meaning. To measure the contextual variability of a particular word, Hoffman et al. calculated the mean distance between all of the contexts that contain the word, thereby providing a measure of relatedness in the contexts across which the word can be used. This quantity was termed a word’s semantic diversity (SemD). Words that tend to appear in a restricted, interrelated set of contexts have low diversity values (e.g., *spinach*, which typically only occurs in contexts related to cooking and eating, has a value of 0.99) and those that appear in a wider range of disparate contexts have high values (e.g., *chance* has a value of 2.08, with the maximum possible values being around 2.4 for function words like *also, which*, and *from*, which can be used in any context).

It is important to note that the SemD measure is not a simple count of the number of contexts containing a given word, as some other researchers have measured (Adelman, Brown, & Quesada, 2006). A contextual frequency count of this kind relates principally to a word’s frequency of use in the language and provides no information as to variation in its meaning across contexts. In contrast, the SemD measure specifically considers the degree of similarity between the various contexts in which a particular word is used (for related approaches, see Jones, Johns, & Recchia, 2012; McDonald & Shillcock, 2001). Importantly, Hoffman et al. found that SemD was positively correlated with the number of senses listed for a word in the Wordnet lexical database (Miller, 1995), but that there was considerable variation in SemD values even for words that had only a single sense. This suggests that even among words traditionally thought of having a single meaning, there can be large variability in contextual usage that could lead to ambiguity in meaning. SemD is also correlated with context availability, a well-established ratings-based measure of the ease with which participants can generate a plausible context when presented with a word (Schwanenflugel, Harnishfeger, & Stowe, 1988; Schwanenflugel & Shoben, 1983). One advantage of the SemD measure over context availability is that it is well-defined and derived directly from linguistic data. In contrast, it is not clear what cognitive processes participants engage in when making context availability judgments, or how directly these ratings relate to the number or breadth of contexts in which words could appear (for further discussion, see Hoffman et al., 2013).

SemD provides a conceptualization of polysemy that rejects the view that word meanings are represented as a discrete number of competing senses. What are the implications of this view for lexical-semantic processing? The effects of word ambiguity on lexical decision have been studied extensively, with many studies reporting a processing *advantage* for more ambiguous words (Azuma & VanOrden, 1997; Borowsky & Masson, 1996; Hino & Lupker, 1996; Jastrzembski, 1981; Piercey & Joordens, 2000; Rubenstein et al., 1970). The direction of this effect may diverge, however, with respect to the nature of the underlying ambiguity. Rodd, Gaskell, and Marslen-Wilson (2002) found while highly polysemous words (defined as those having many senses in the dictionary) were processed more quickly than nonpolysemous words, there was a *disadvantage* for homonymous words with multiple unrelated meanings. The authors later simulated their findings in a connectionist computational model that used variable, distributed semantic representations (Rodd et al., 2004). The model mapped from orthographic inputs onto semantic units. Polysemous words were assumed to have variable semantic representations, such that the same word could be mapped to a number of distinct but related semantic patterns. The authors did not specify how this variation came about; however, on the SemD view, this is assumed to be a direct consequence of the word being used in a wide variety of contexts. In any case, as a consequence of this variability, each polysemous word developed a broad attractor basin within the semantic network and was quick to settle into this semantic neighborhood when its orthographic form was presented. In contrast, words with no semantic variability were associated with narrower attractor basins and settled more slowly in the initial stages following word presentation. This difference in initial settling speed over the semantic layer was held to explain the advantage for polysemous words in lexical decision (for a related account, see Armstrong & Plaut, 2008). This account predicts similar effects for words high in SemD, because these are also thought to have variable semantic representations as a consequence of being used in a range of disparate contexts. In Experiment 1, we tested this prediction.

Semantic ambiguity typically has a negative effect on tasks that require comprehension or explicit semantic processing (Balota & Paul, 1996; Duffy, Morris, & Rayner, 1988; Hino, Pexman, & Lupker, 2006; Pexman, Hino, & Lupker, 2004). In particular, a number of studies have investigated ambiguity effects (typically using homonyms) in the relatedness decision task, in which participants judge whether two words are related to one another in meaning (Gottlob, Goldinger, Stone, & Van Orden, 1999; Pexman et al., 2004; Piercey & Joordens, 2000). The principal finding of these studies is that participants are slower to verify that two words are related when one of the words is ambiguous (e.g., vampire–bat). This effect has been explained in two ways. Some researchers have attributed it to slower activation of the correct semantic pattern for ambiguous words (Piercey & Joordens, 2000; Rodd et al., 2004). In Rodd et al.’s model, for example, although the semantic representations of polysemous words initially settle more quickly, this is followed by a later phase in which both polysemous and homonymous words settle more slowly than their unambiguous counterparts. Other authors have attributed the ambiguity disadvantage to conflict at the decision-making stage of processing (Hino et al., 2006; Pexman et al., 2004). Taking the vampire–bat example, they assume that both interpretations of *bat* are simultaneously activated when the word is processed. The *bat = flying mammal* interpretation signals a “yes” response while the *bat = sports equipment* interpretation simultaneously signals a “no” response. There is therefore response competition which delays the production of a response. Critically, this account predicts that no such disadvantage should be observed for unrelated word pairs (e.g., sandwich–bat) because both interpretations signal a “no” response. In line with this prediction, Pexman, Hino, and Lupker (2004) found that while responses to related word pairs were slower when one of the words was a homonym, there was no such effect for unrelated word pairs.

The competition explanation is particularly suitable for homonyms, for which it is reasonable to assume that there are two unrelated interpretations that compete for selection. In contrast, another explanation may be needed for high SemD words, whose ambiguity is thought to come from graded, context-dependent variation in meaning. High SemD words occur in many different linguistic contexts, with concomitant variation in the semantic activation elicited. As a consequence, when a high SemD word is encountered in a weak or novel context there is uncertainty about exactly what form the semantic activation should take. Under these conditions, the semantic system may settle into a noisy, somewhat underspecified state that represents a blend of the possible semantic patterns associated with the word. In contrast, low SemD words settle into more stable states because there is more consistency in their semantic patterns across contexts. We predict that the additional uncertainty in the semantic pattern of a high SemD word means that when it is compared with another word, it takes longer to determine whether their patterns are related. Importantly, this explanation may hold irrespective of whether the eventual response is “yes” or “no.” In Experiment 2, we tested this prediction. We also provide a formal connectionist simulation of these ideas.

The main aim of the study, therefore, was investigate the effects of SemD on lexical-semantic processing using lexical decision (Experiment 1) and relatedness decision (Experiment 2) tasks. Yet SemD naturally covaries with another factor, imageability. Imageability refers to the degree to which a word elicits mental imagery and has a well-established facilitatory effect on processing in lexical decision (e.g., Evans, Lambon Ralph, & Woollams, 2012; James, 1975) and in comprehension tasks (Holmes & Langford, 1976; Schwanenflugel & Shoben, 1983; Yap, Pexman, Wellsby, Hargreaves, & Huff, 2012). This effect has been explained either in terms of highly imageable words having richer semantic representations (Plaut & Shallice, 1993; Yap et al., 2012) or in terms of less imageable words being more contextually variable (Schwanenflugel et al., 1988; Schwanenflugel & Shoben, 1983). In previous work we found a negative correlation between imageability and SemD (Hoffman et al., 2013), supporting the view that less imageable words have more variable, context-dependent meanings. Previous reports of imageability effects in lexical-semantic tasks may therefore have also reflected an influence of SemD, at least in those situations in which SemD would be expected to have a negative influence on performance. Hence, we included an orthogonal manipulation of imageability in both experiments, allowing us to investigate whether imageability effects would still be observed when SemD was controlled.

## Experiment 1: Lexical Decision

### Method

#### Participants

Twenty-four undergraduate students at the University of Manchester took part in exchange for course credit (23 female; mean age = 19.2). All were native English speakers.

#### Materials

We began by creating four sets of 60 monosyllabic words that varied imageability and SemD in an orthogonal fashion. The properties of the four sets are shown in Table 1, with the items provided in the Appendix. Imageability values were obtained from the Cortese and Fugett (2004) database and SemD values were calculated based on the British National Corpus (BNC, 2007), following the method described by Hoffman, Lambon Ralph, and Rogers (2013). A 2 × 2 ANOVA confirmed that high and low imageability words differed in their imageability values, *F*(1, 240) = 1236, *p* < .001 but that high versus low SemD words did not, *F*(1, 240) = 1.23, *p* = .27. Likewise, high and low SemD words differed in SemD, *F*(1, 240) = 608, *p* < .001 but high and low imageability words did not, *F*(1, 240) = 0.67, *p* = .42. Stimulus selection was aided by the Match program (van Casteren & Davis, 2007). Stimuli in the four conditions were matched for the following variables: word length, log word frequency in the BNC, subjective frequency ratings reported by Balota, Pilotti, and Cortese (2001), bigram frequency and number of orthographic neighbors (both obtained using the N-watch application; Davis, 2005) and age of acquisition ratings reported by Cortese and Khanna (2008), all *F* < 1.[Table-anchor tbl1]

In this study, we were principally interested in ambiguity effects related to polysemy and not to homonymy. The SemD measure makes no distinction between these types of ambiguity and simply measures the degree of variability between the contexts in which a word is used. The stimulus set therefore contained a mixture of homonyms and nonhomonymous words. Table 1 shows the proportion of homonyms in each condition, where a homonym was defined as any word with more than one lexical entry in the Wordsmyth online dictionary (cf. Rodd et al., 2002). The rates did not differ across conditions (high vs. low SemD: χ^2^ = 0.32, *p* = .71; high vs. low imageability: χ^2^ = 1.74, *p* = .19). However, to ensure that homonym status could not account for our results, we included a binary classification of homonymy as a covariate in all item analyses.

We constructed 240 nonword foils by first selecting 240 real words that were matched to the targets for length, word frequency and bigram frequency on a pairwise basis. We then added, altered or removed one or two letters of each of these words to form pronounceable nonwords, the properties of which are shown in Table 1. The nonwords did not differ from the word stimuli on bigram frequency, *t*(478) = 1.23, *p* = .22, though they were slightly longer, *t*(478) = 2.11, *p* = .04 and had fewer orthographic neighbors, *t*(478) = 2.39, *p* = .02.

#### Procedure

Participants made lexical decisions to all 240 word targets and 240 nonwords, presented in a random order. Each trial began with a fixation cross, presented for 500 ms, followed by the stimulus, presented in black text on a white background (36 point Arial font). Responses were collected via button box and the experiment was preceded by a practice block of 20 trials.

#### Data analysis

Each participant’s data were first screened to exclude participants with poor or inattentive performance. One participant was removed due to taking more than 2 s to respond on more than 5% of trials. Each participant’s data were then trimmed by removing any RTs that fell more than two standard deviations outside their overall mean. Error rates and RTs were considered as dependent measures and data were analyzed separately by subjects (*F*_1_) and by items (*F*_2_).

### Results

#### Error rates

**Figure 1 fig1:**
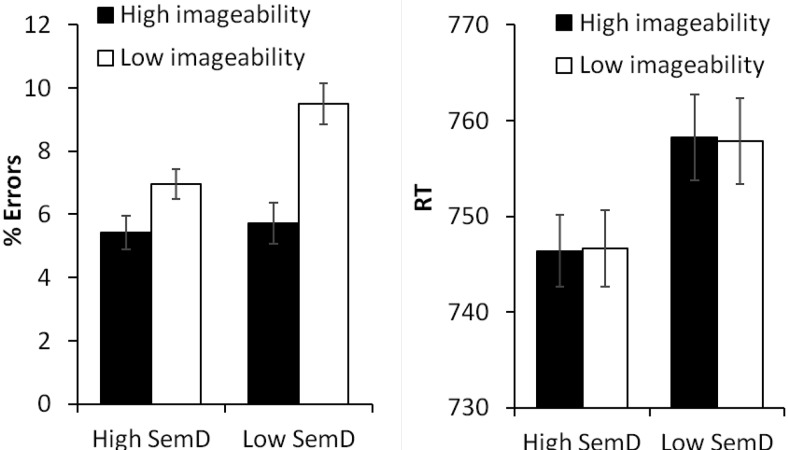
Results for lexical-decision task (Experiment 1) according to imageability and semantic diversity. Bars indicate standard error of mean, adjusted to reflect the between-condition variance used in repeated-measure designs (Loftus & Masson, 1994).

Results are presented in Figure 1. Responses to words were analyzed in 2 (SemD) × 2 (imageability) ANOVAs. These revealed a main effect of imageability, *F*_1_(1,22) = 13.5, *p* = .001; *F*_2_(1,235) = 4.74, *p* = .03, and a main effect of SemD that was only significant by subjects, *F*_1_(1,22) = 5.93, *p* = .023; *F*_2_(1,235) = 1.28, *p* = .26. Participants made an additional 1.4% errors for low SemD words relative to high SemD, while low imageability words elicited 2.7% more errors than high imageability words. The interaction between the two factors fell short of statistical significance, *F*_1_(1,22) = 2.74, *p* = .11; *F*_2_(1,235) = .84, *p* = .36.[Fig-anchor fig1]

#### Reaction times

There was a main effect of SemD that was highly significant by subjects and marginal by items, *F*_1_(1,22) = 9.99, *p* = .005; *F*_2_(1,235) = 2.80, *p* = .096: Responses to high SemD words were on average 12 ms faster than responses to low SemD words. There was no effect of imageability and no interaction. As the effect of SemD was weak in the by-items analysis, we investigated this effect further by constructing a multiple regression model that included RT as the dependent variable and all of the variables listed in Table 1 as predictors. In this model, there was a significant effect of SemD (β = −.11, *t* = 2.04, *p* = .04), supporting the finding that SemD had a facilitatory effect on RT.

### Discussion

In lexical decision, words with high SemD were responded to 12ms faster than words of low SemD. This establishes that the SemD measure yields a similar ambiguity advantage in this task as polysemous words selected based on number of dictionary definitions or distinct meanings generated by participants (Azuma & VanOrden, 1997; Borowsky & Masson, 1996; Hino & Lupker, 1996; Jastrzembski, 1981; Piercey & Joordens, 2000; Rubenstein et al., 1970). The precise mechanism underlying this advantage varies across models of lexical decision. Some authors have assumed that decisions are made based on the strength of semantic activation elicited by the stimulus (Armstrong & Plaut, 2008; Borowsky & Besner, 1993; Plaut, 1997; Rodd et al., 2004). These models produce an advantage for ambiguous words because these words generate stronger initial semantic activation. We provide a simulation of this effect for high and low SemD words later in the article. In other models of lexical decision, the critical factor is the degree to which the orthographic activity elicited resembles that of a real word (Dilkina, McClelland, & Plaut, 2010; Harm & Seidenberg, 2004; Pexman & Lupker, 1999). Because of interactivity between levels of representation, semantic activation is an important source of feedback, helping the orthographic pattern to settle more quickly. These models could also account for the ambiguity advantage in terms of stronger initial semantic activation.

Consistent with previous studies (Cortese & Khanna, 2007; Evans et al., 2012; James, 1975), we also found that highly imageable words were processed more efficiently, resulting in fewer errors. Although there was a tendency for this effect to be stronger for low SemD words, consistent with previous research showing imageability effects only for unambiguous words (Tokowicz & Kroll, 2007; but see Rubenstein et al., 1970), the interaction was not significant. The presence of imageability effects for both high and low SemD words indicate that accounts of this dimension purely in terms of context availability/variability are unlikely to be sufficient. These results are, however, in keeping with the commonly held view that highly imageable words have richer semantic representations (Wiemer-Hastings & Xu, 2005; Plaut & Shallice, 1993) and that more robust activation within the semantic system leads to more efficient lexical decision, particularly when foils cannot be rejected on the basis of subword orthographic structure alone (e.g., Chang, Lambon Ralph, Furber, & Welbourne, 2013; Evans et al., 2012; Pexman, Hargreaves, Siakaluk, Bodner, & Pope, 2008). Here, we established that this effect still holds when controlling for the greater contextual variability of less imageable words.

The imageability effect was weaker, however, than that seen in previous studies. There most likely explanation for this stems from the fact that semantic effects in lexical decision are highly dependent on the properties of the nonword foils, increasing as foils become harder to distinguish from real words (Evans et al., 2012). Our nonwords had significantly fewer orthographic neighbors than our words and this difference may have reduced participants’ reliance on semantic factors. In addition, our stimuli did not vary imageability as strongly as some previous studies (e.g., Evans et al., 2012) and this may have affected our ability to detect imageability effects.

## Experiment 2: Semantic Relatedness Judgments

In Experiment 1, we observed an advantage for high SemD words and highly imageable words in lexical decision. In Experiment 2, we investigated the effects of SemD and imageability during semantic relatedness judgments. An ambiguity disadvantage has been reliably observed in this task on trials where the two words share a semantic relationship (“yes” trials) but has not been found on “no” trials consisting of unrelated words (Gottlob et al., 1999; Pexman et al., 2004; Piercey & Joordens, 2000). The main aim of the experiment was to test whether similar effects would emerge under our alternative method of considering ambiguity as a function of contextual variability. We also controlled for imageability, as this dimension has not been taken into account in previous work. An imageability advantage has been observed in a number of different semantic processing tasks (Hoffman et al., 2013; Holmes & Langford, 1976; Jefferies, Patterson, Jones, & Lambon Ralph, 2009; Schwanenflugel & Shoben, 1983) but has yet to be investigated in the relatedness decision task. Here, using a factorial manipulation, we investigated whether an imageability effect would be observed in this task when controlling for SemD.

### Method

#### Participants

Twenty-five undergraduate students at the University of Manchester took part in exchange for course credit (19 female; mean age = 20.6). All were native English speakers.

#### Materials

Participants made semantic relatedness judgments to sequentially presented word pairs. The 240 words from Experiment 1 served as the second word in each pair. For the first word in each pair, we selected a new word that shared a semantic relationship with the second. We adopted an inclusive definition of semantic relationships, which included shared category membership (e.g., stomach–chest), synonymy and antonymy (e.g., slept–woke), semantic association (e.g., thirst–drought) and action-recipient relationships (e.g., roll–dice). First word properties are listed in Table 2, with the items provided in the Appendix. Imageability and SemD were manipulated for the first words as well as the second. The first words on high imageability trials were more imageable than those for low imageability trials, *F*(1, 240) = 310, *p* < .001 and there was a significant difference in SemD between high and low SemD first words, *F*(1, 240) = 100, *p* < .001. By applying imageability and SemD manipulations to both words in each pair, we aimed to maximize the strength of these manipulations. However, one limitation of this design choice is that we were unable to determine whether experimental effects were due to the properties of the first word or second word in the pair, or some combination of the two.[Table-anchor tbl2]

First words were matched across conditions for word length, log word frequency, syllable length, bigram frequency and number of neighbors (*F* < 2.3, *p* > .13). To quantify the strength of the relationship between word pairs, we consulted a large database of free association norms (Nelson, McEvoy, & Schreiber, 1998); 90% of the first words were present in the database. For each first word, we calculated the percentage of participants who produced our second word in response to the first. The mean production rates, shown in Table 2, were low but did not differ across conditions (*F* < 1).

To form unrelated word pairs, first words were randomly assigned to a different second word within the same condition, and we then checked that there was no relationship between the words in the new pairings.

#### Procedure

Participants made semantic relatedness judgments to 240 word pairs. On half of the trials, the first word was related to the second and on the remaining trials it was unrelated. Each participant saw all 240 second words, with the first words counterbalanced such that each second word was seen with a related word by half of the participants and with an unrelated word for the other half. Note, however, that data from an odd number of participants was entered into the final analysis, so this factor could not be entirely counterbalanced. There was no repetition of words within participants.

Each trial began with a fixation cross, presented for 500 ms. The first word was then presented for 1,000 ms, in black text on a white background (36 point Arial font), and was immediately followed by the second. Participants were asked to decide whether the second word was related in meaning to the first, responding by pressing one of two buttons. The experiment was preceded by a number of examples of related and unrelated pairs and by a practice block of 20 trials.

#### Data analysis

Each participant’s data were screened to exclude participants with poor or inattentive performance. Two participants were excluded as they made more than 30% errors on either related or unrelated trials. Reaction times falling more than two standard deviations from a participant’s overall mean were again removed. Error rates and RTs were considered as dependent measures.

### Results

#### Error rates

**Figure 2 fig2:**
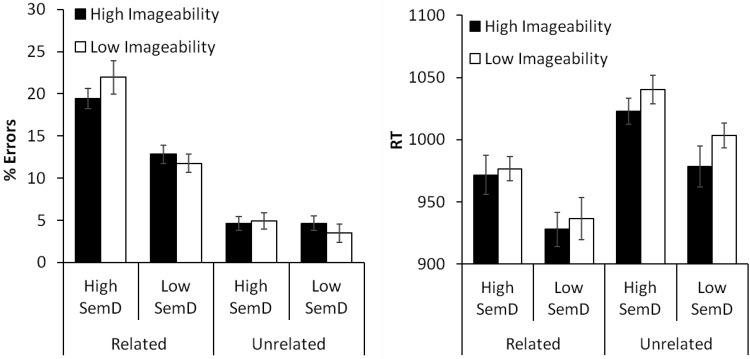
Results for semantic relatedness decision task (Experiment 2) according to imageability, semantic diversity and relatedness. Bars indicate standard error of mean, adjusted to reflect the between-condition variance used in repeated-measure designs (Loftus & Masson, 1994).

Results for the experiment are shown in Figure 2. Data were analyzed in a 2 × 2 × 2 ANOVA that included imageability, SemD, and relatedness as within-subjects factors. There was no effect of imageability, *F*_1_(1,22) = 0.04 *p* = .85; *F*_2_(1,235) = 0.03, *p* = .86; however, there were main effects of SemD, *F*_1_(1,22) = 45.4, *p* < .001; *F*_2_(1,235) = 10.6, *p* = .001; and relatedness, *F*_1_(1,22) = 69.0, *p* < .001; *F*_2_(1,235) = 50.7, *p* < .001; as well as an interaction between these factors, *F*_1_(1,22) = 36.4, *p* < .001; *F*_2_(1,235) = 8.02, *p* = .005. Further analyses revealed that, for related word pairs, participants made more errors on high SemD trials than low SemD trials, *F*_1_(1,22) = 60.5, *p* < .001; *F*_2_(1,235) = 11.3, *p* = .001; while there was no effect of SemD for unrelated pairs, *F*_1_(1,22) = 0.93, *p* = .35; *F*_2_(1,235) = 0.12, *p* = .55.[Fig-anchor fig2]

#### Reaction times

There were main effects of relatedness, *F*_1_(1,22) = 10.9, *p* < .001; *F*_2_(1,235) = 19.8, *p* < .001; and SemD, *F*_1_(1,22) = 33.2, *p* < .001; *F*_2_(1,235) = 13.3, *p* < .001. In addition, the effect of imageability was significant by subjects only, *F*_1_(1,22) = 4.66, *p* = .042; *F*_2_(1,235) = 1.37, *p* = .24. There were no interactions. On average, decisions to high SemD word pairs were 41 ms slower than decisions to low SemD pairs and responses to highly imageable words were 14 ms faster than those to less imageable words. Responses were 58 ms faster on related trials compared with unrelated trials. As the effect of imageability was weak in the by-items analysis, we investigated this effect further by again constructing a multiple regression model that included RT as the dependent variable and all of the variables listed in Table 1 as predictors. The model indicating a significant effect of imageability (β = −.13, *t* = 1.99, *p* = .047), supporting the finding that imageability had a facilitatory effect on RT. We also analyzed data for the related and unrelated pairs separately. There were significant effects of SemD for both related pairs, *F*_1_(1,22) = 9.03, *p* = .007; *F*_2_(1,235) = 5.34, *p* = .022, and unrelated pairs, *F*_1_(1,22) = 19.9, *p* < .001; *F*_2_(1,235) = 5.27, *p* = .023.

### Discussion

In contrast to the advantage for high SemD words observed in lexical decision (Experiment 1), here we found substantially slower responses for high SemD words. This main effect is similar to the ambiguity disadvantage observed previously in this task (Gottlob et al., 1999; Pexman et al., 2004; Piercey & Joordens, 2000). We also found a weak effect of imageability on RT, favoring highly imageable words, though this was only significant over participants, not items. Other studies have found more robust imageability effects in semantic processing tasks (Hoffman et al., 2013; Holmes & Langford, 1976; Jefferies et al., 2009; Schwanenflugel & Shoben, 1983). The weak effect observed here could be explained in two ways. First, with one exception (Hoffman et al., 2013), previous studies have not controlled for SemD. Imageability is negatively correlated with SemD, so previous findings of imageability effects may be partly attributable to the negative effects of SemD on semantic processing. Second, the size of the imageability effect may be task-dependent. Previous studies have employed either sentence processing tasks (Holmes & Langford, 1976; Schwanenflugel & Shoben, 1983) or multiple-alternative choice tasks (Hoffman et al., 2013; Jefferies et al., 2009) which are more complex and may therefore be more sensitive to the imageability of the words probed.

Pexman et al. (2004) found that the processing disadvantage for ambiguous words is confined to decisions made on related word pairs. This result has been explained in terms of response conflict on related trials, arising from the fact that one interpretation of the ambiguous word is related to the other word in the pair but other interpretations are not. On this view, no such conflict occurs on unrelated trials because all interpretations of the ambiguous word are unrelated to the other word. In contrast, here we found that high SemD words were at a disadvantage irrespective of whether they appeared in related or unrelated word pairs. There are two experimental factors that may explain the divergent results. The first is that we manipulated ambiguity in both words in the pair (i.e., in our high SemD condition, both words were high in SemD) while previous studies used pairs comprising one ambiguous and one unambiguous word. This may have given us greater power to detect effects of semantic ambiguity. The second, and more important, factor is that Pexman et al. (2004) focused on homonyms with two distinct meanings, while the SemD measure employed here indexes graded, polysemous semantic variation. Pexman et al. selected ambiguous words that were judged by participants to have more than one meaning. It is likely that these judgments primarily reflect homonymy, as this is the most salient form of semantic ambiguity for participants without specific linguistic training. To explore this possibility, we obtained SemD values for the stimuli that produced a null effect of ambiguity on “no” trials in Pexman et al.’s study (their Experiment 2). The ambiguous and unambiguous words in this experiment did not differ in their mean SemD values (means of 1.71 vs. 1.75; *t* = 0.6, *p* = .55). This indicates that Pexman et al. were measuring a different form of ambiguity to that indexed by SemD.^1^ The ambiguous words in the Pexman et al. study typically had two distinct meanings and were therefore likely to suffer from competition between these interpretations. However, a different explanation is required for the SemD effects observed in the present study.

We propose that slower “yes” and “no” decisions to high SemD words are best understood by considering the greater variability in the semantic patterns of these words and the effect that this has on the time taken to decide whether the word pairs are related. In the decision task, there is no strong context to guide how a particular word should be interpreted. As a result, the semantic activations elicited will tend to an average or blending of all the semantic states with which the word has been associated in the past (Borowsky & Masson, 1996; Piercey & Joordens, 2000). Because there is greater variability in the past states of high SemD words, their blend states are more noisy or less well-specified than the blend states of low SemD words. We propose that this additional noise slows decisions to word pairs of this type, irrespective of whether the words are semantically related. In the next section, we provide a simulation of this effect in a connectionist computational model.

## Connectionist Simulation of Meaning Activation for High and Low SemD Words

We simulated relatedness decisions in a connectionist model that was trained to activate distributed semantic representations for individual words when presented with orthographic input patterns. The architecture was based on previous models that have simulated ambiguity effects in lexical decision (Armstrong & Plaut, 2008; Rodd et al., 2004). In these models, each polysemous word was associated with a number of distinct but related semantic patterns, generated by distorting a central prototype. This reflects the idea that the meanings of polysemous words change when they are used in different contexts but that the various uses are related to one another in meaning. Here, we made a similar assumption about high SemD words. Our model differed from those of Armstrong and Plaut (2008) and Rodd, Gaskell, and Marslen-Wilson (2004) in two important ways. First, we treated ambiguity as a continuous property that is true of all words to varying extents. Consequently, both high and low SemD words mapped to multiple, variable semantic patterns and the greater variability of high SemD words was modeled by distorting their patterns more strongly from their central prototype. Second, while previous models were concerned with lexical decision, our focus was on semantic relatedness decisions. However, we did also explore whether our model could replicate the finding of greater semantic activation for highly polysemous words, which is thought to underpin their advantage in lexical decision.

### Method

#### Model architecture

**Figure 3 fig3:**
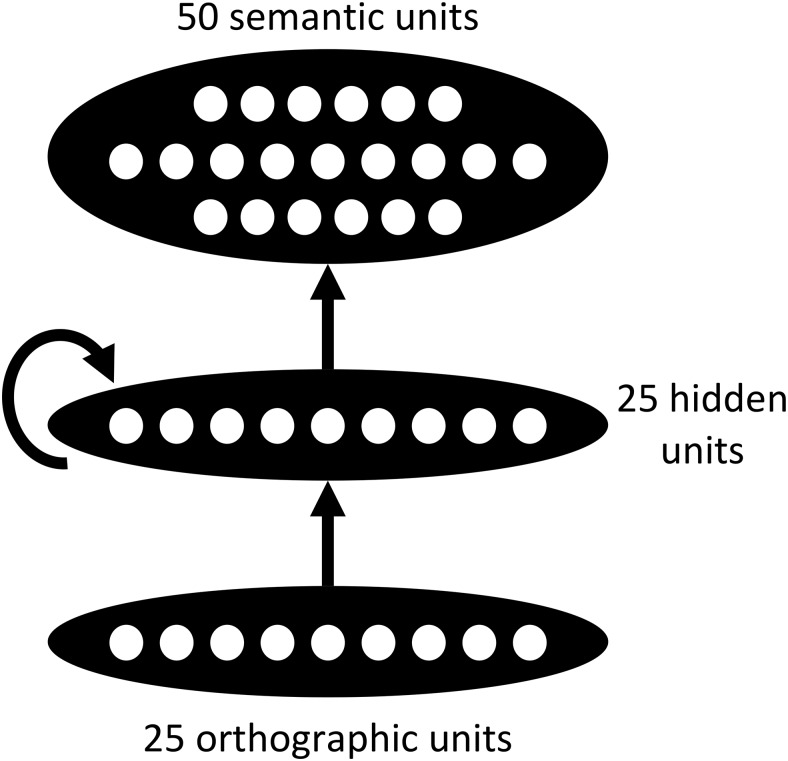
Architecture of the connectionist simulation.

The model consisted of 25 orthographic input units and 50 semantic units, with connections between them mediated by 25 hidden units (see Figure 3). There were feed-forward projections from the orthographic units to the hidden units and from the hidden units to the semantic units. In addition, hidden units were fully connected with one another. Activation of units ranged between 0 and 1 and was computed according to a logistic function. Hidden units and semantic units also received fixed bias inputs of −5, which meant that in the absence of any other input they would remain close to their minimum activation level.[Fig-anchor fig3]

#### Orthographic and semantic representations

Orthographic and semantic patterns were generated for 48 low SemD words and 48 high SemD words. Orthographic patterns followed a simple consonant-vowel-consonant (CVC) structure. Ten orthographic units represented initial consonants, five vowels and 10 final consonants. Each word was randomly assigned a unique orthographic pattern in which one initial consonant, one vowel and one final consonant were activated. In common with most connectionist approaches to semantic representation (e.g., Plaut, 1997), semantic representations were implemented as abstract distributed patterns of activation over the semantic layer. On this view, individual semantic units can be thought of as representing semantic features which may be present or absent for particular words. On this view, the semantic relatedness of two words is determined by the degree to which they activate the same semantic units.

Each word was randomly assigned a prototype semantic pattern in which exactly 25 of the 50 semantic units were activated. Prototypes were generated such that each word had (a) a semantically related word with which it shared 20 activated units, and (b) an unrelated word with which it shared only five activated units.^2^ High and low SemD words did not differ in the number of activated units in their prototype, or in the degree of overlap between the prototypes of related or unrelated word pairs. They did differ, however, in the degree to which their semantic patterns deviated from the prototype on each presentation, as we describe next.

#### Training

On each trial, the network was presented with the orthographic pattern for a particular word and trained to produce an appropriate activation pattern over the semantic layer. Processing took place over seven time intervals, each divided into four time steps or ticks. The orthographic units were hard-clamped with the pattern for a particular word throughout this period and activation was allowed to cycle through the rest of the network. On the final two intervals of processing, the appropriate semantic pattern was applied as a target to the semantic units. Error was computed by comparing the actual activation of the semantic units with their targets and connections throughout the network were adjusted using the “back-propagation through time” algorithm (Rumelhart, Hinton, & Williams, 1986). This process made small, incremental changes to the connection weights such that over many learning experiences the network came to reliably activate a particular semantic pattern in response to each orthographic pattern.

Importantly, the semantic targets changed slightly each time a given word was presented to the network. This variation reflects our view that the semantic information associated with individual words varies as a function of context each time the word is encountered. Specifically, we generated 50 different semantic target patterns for each word by distorting its prototype pattern. To distort high SemD words, each unit in the prototype had a probability of 0.2 of changing from its original value (from 0 to 1 or vice versa). For low SemD words, the probability was 0.1. This reflects the idea that all words are associated with some degree of semantic variation but that this variation is greater for high SemD words because they occur in a wider variety of contexts. Each time a particular word was presented to the network, one of its 50 target patterns was selected at random and applied to the semantic layer.

Other parameters of the network were as follows. The learning rate was 0.2 and momentum of 0.9 was applied only when the gradient of the error slope was less than 1. Weight decay of 10^−6^ was applied to all connections. Weight updates occurred after every 96 word presentations and training proceeded for a total of 1,000 updates. To encourage the network to activate all of the semantic information associated with a word, a constant of 0.5 was added to all active targets. Simulations were performed using LENS software (www.stanford.edu/group/mbc/LENSManual/index.html).

We trained 10 separate networks in this way, each using the same parameters and training patterns but initialized with different random starting weights. This allowed us to test whether effects were reliable across models (*F*_1_) and whether they were reliable across words (*F*_2_).

#### Testing

To simulate relatedness decisions, we presented the network with two words sequentially and compared the semantic activations elicited by each. The orthographic pattern for the first word in the pair was presented initially, the network was allowed to settle for the full seven processing intervals and the final activation pattern across the semantic units was recorded and stored. Next, the second word in the pair was presented. After each time-step of processing the second word, the current activation pattern on the semantic layer was compared with the stored pattern elicited by the first word. Similarity between the two patterns was quantified by computing the Pearson’s correlation between their activation vectors. We assumed that a “yes” response would be triggered once the similarity between the two patterns exceeded an upper threshold and that a “no” response would occur if the similarity fell below a lower threshold.

Though our main focus was on relatedness decisions, we also considered whether the network could replicate the advantage for polysemous words in lexical decisions, previously demonstrated in similar models (Armstrong & Plaut, 2008; Rodd et al., 2004). Following those models, we did not implement a formal mechanism for making lexical decisions, but rather assumed that greater activation of the semantic units would result in faster lexical decisions. We therefore presented each word to the network in turn and recorded, at each time-step, the total activation it elicited over the semantic layer.

### Results

During training, the network learned to associate the same, fixed orthographic inputs with variable semantic patterns. The consequence of this variability was that the network learned to activate a composite semantic pattern for each word that represented a blend of all the different patterns it had experienced for the word. Because high SemD words were associated with greater variability, their composite semantic patterns were more noisy (i.e., individual units often adopted activation values in the middle of their range and were less likely to adopt extreme values close to 0 or 1).

#### Similarity between word pairs

**Figure 4 fig4:**
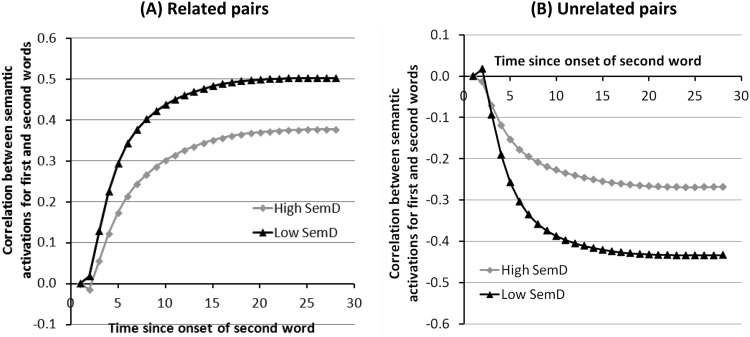
Similarity of semantic patterns for related and unrelated word pairs.

To simulate relatedness decisions, we monitored the activation on the semantic layer during processing of the second word in the pair and compared this with the final activation state elicited by the first word. The results of these comparisons are shown in Figure 4. For related pairs, the initial correlation was near zero, but as the semantic pattern for the second word settled, it became more similar to the pattern for the first word. Importantly, the similarity was greater for low SemD pairs. We investigated this effect by conducting 2 (Word Type) × 28 (Time-Step) ANOVAs on the data. As expected, there was a main effect of SemD, *F*_1_(1,9) = 66.8, *p* < .001; *F*_2_(1,94) = 23.0, *p* < .001. There was also a main effect of time, *F*_1_(27,243) = 828, *p* < .001; *F*_2_(27,2538) = 684, *p* < .001; and an interaction, *F*_1_(27,243) = 13.4, *p* < .001; *F*_2_(27,2538) = 9.9, *p* < .001; indicating that the SemD effect grew as processing progressed. Though we did not implement a specific response generation mechanism, we assumed that a “yes” response would be triggered when similarity exceeded a threshold value. It is clear from Figure 4 that wherever this threshold was set, low SemD words would reach it before high SemD words.[Fig-anchor fig4]

The reverse pattern was observed for unrelated word pairs. Here, the two semantic patterns became more dissimilar throughout processing, with the level of dissimilarity being greater for low SemD words. This was confirmed by 2 × 28 ANOVAs, which revealed effects of SemD, *F*_1_(1,9) = 1927, *p* < .001; *F*_2_(1,94) = 80.7, *p* < .001; time, *F*_1_(27,243) = 67.3, *p* < .001; *F*_2_(27,2538) = 616, *p* < .001; and an interaction, *F*_1_(27,243) = 1149, *p* < .001; *F*_2_(27,2538) = 42.5, *p* < .001. We assumed that a “no” response would be triggered when the dissimilarity exceeded a negative threshold; it is clear from the figure that the threshold would be reached more quickly for low SemD words.

#### Speed and magnitude of semantic activation

**Figure 5 fig5:**
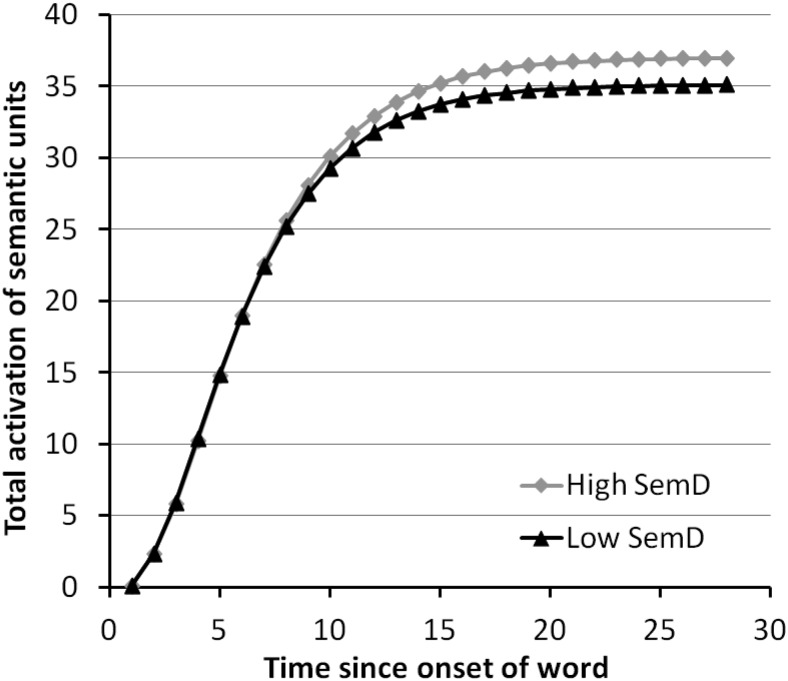
Total activation of semantic units during word processing.

It is generally assumed that more rapid and robust activation of semantic representations has a beneficial effect on lexical decisions. We would therefore expect the model to produce greater activation for high SemD words, since Experiment 1 showed a small RT advantage for these words in lexical decision. Figure 5 shows the total activation of the semantic units at each time-step for high and low SemD words. We analyzed these results with 2 (Word Type) × 28 (Time-Step) ANOVAs. There was a main effect of SemD, *F*_1_(1,9) = 254, *p* < .001; *F*_2_(1,94) = 158, *p* < .001; favoring high SemD words, an effect of time, *F*_1_(27,243) = 3331, *p* < .001; *F*_2_(27,2538) = 120,812, *p* < .001; and an interaction, *F*_1_(27,243) = 226, *p* < .001; *F*_2_(27,2538) = 152, *p* < .001. This confirms that high SemD words elicited more rapid and robust activation of the semantic units overall, indicating that the model could account for the processing advantage for these words in lexical decision.[Fig-anchor fig5]

### Discussion

Our simulation of SemD effects in relatedness decisions was based on the principle that the semantic representations of words vary across contexts. When a word is presented without a strong context, the semantic activation generated represented a composite of these previous semantic associations. Because high SemD words are associated with greater contextual variability, their composite activations were more noisy and this had two consequences for decisions. First, the activation patterns of semantically related high SemD words were less similar to one another than those of low SemD words, resulting in slower decisions for these on “yes” trials. Second, for high SemD unrelated word pairs, the two representations were less differentiated from one another than for low SemD, resulting in slower “no” decisions. We also investigated total activation over the semantic units. We found that high SemD words generated activation more quickly than low SemD words, indicating that the model could also account for faster lexical decision times to these words.

## General Discussion

Psycholinguistic studies have frequently considered the effects of homonymy and polsysemy on lexical and semantic processing (for a recent review, see Eddington & Tokowicz, 2015). Many studies have reasonably concluded that homonyms (e.g., *bark*) have two distinct semantic representations that compete with one another for activation when the word is processed (Jastrzembski, 1981; Kellas et al., 1988; Morton, 1979; Rubenstein et al., 1970). Some authors have claimed that the senses of polysemous words (e.g., *chance*) are represented in the same way (Klein & Murphy, 2001). Many more adhere to the view that all uses of a polysemous word can be classified as one of a small number of discrete but related senses (e.g., Azuma & VanOrden, 1997; Borowsky & Masson, 1996; Jastrzembski, 1981; Rodd et al., 2002). In this study, we used an alternative approach to define polysemy, based on the idea that polysemous variation in word meaning is a continuous, graded phenomenon that is present for words to differing degrees as a function of their contextual variability. We quantified this variability using semantic diversity (SemD), a measure that assesses the level of semantic similarity among the various contexts that contain a particular word (Hoffman et al., 2013). We found that participants showed a beneficial effect of greater SemD in lexical decision, in line with previous findings for polysemous words (Azuma & VanOrden, 1997; Hino et al., 2006; Klepousniotou & Baum, 2007; Rodd et al., 2002). When making semantic relatedness decisions, however, participants were slower to respond to pairs of high SemD words, similar to the SemD effect seen in healthy older participants in a synonym judgment task (Hoffman et al., 2013). Importantly, the effect we observed in relatedness decision held for related word pairs on which a “yes” response was required but also on “no” trials composed of unrelated words. This result contrasts with that found previously for homonyms, in which an ambiguity disadvantage was only observed on “yes” trials (Pexman et al., 2004), and suggests that different mechanisms are at play for different types of semantic ambiguity.

Pexman et al. (2004) explained their results in terms of response conflict that occurs on “yes” trials, when one interpretation of the ambiguous word is related but the other is not. In contrast, no conflict was thought to occur on “no” trials. While this account is plausible for words with opposing, homonymous meanings, an alternative explanation is required for our finding of slower performance for high SemD words for “yes” and “no” responses. Our explanation is based on the proposal that, unlike homonyms, the ambiguity associated with high SemD words is best thought of as graded, context-dependent variation around a central semantic representation. We simulated our findings in a connectionist computational model that learned to map from orthographic inputs patterns to distributed semantic representations. The greater variability in the semantic representations of high SemD words led these words to be associated with noisier semantic patterns. As a consequence, the patterns of related high SemD words were less similar to one another, and unrelated words more poorly distinguished from one another, compared with low SemD words. It is this noisy instantiation of the semantic patterns of high SemD words that we claim accounts for the behavioral decrements for these words in the relatedness judgment task.

Our account differs from that of Pexman et al. (2004) in that we predict SemD effects for both related and unrelated trials. Pexman et al. assumed that any conflict between meanings is irrelevant if the ambiguous word is being compared with a word that is unrelated to all of its meanings. We argue instead that when a word is associated with a wide variety of different contextual uses, the word develops an intrinsically noisy semantic representation and this affects processing for *all* comparisons, even when comparing with words with very distinct semantic representations. That said, the two theories share the principle that ambiguity disadvantages in relatedness decision arise from difficulties in making the relatedness decision, rather than differences in the speed with which the meanings of ambiguous and unambiguous words are activated. In this respect, our account differs from that presented by Rodd et al. (2004; see also Armstrong & Plaut, 2008). These authors have argued that ambiguity effects can be explained entirely in terms of the time-course of semantic activation. In their simulations, polysemous words elicit rapid initial semantic activation but take longer to settle into their final activation states. As a consequence, lexical decisions, which can be made on the basis of early semantic activation, are faster for polysemous words but decisions that require access to a more settled semantic pattern (e.g., relatedness judgments) are slower (for similar arguments, see Piercey & Joordens, 2000). Our model also assumes that the SemD advantage in lexical decision results from faster initial semantic activation. However, our explanation of SemD effects in relatedness decision is based on the noisiness of the semantic representations and not on the speed with which they become active.

We also found a significant effect of imageability on accuracy in lexical decision for both high and low SemD words. Previous research on the nature of the interaction between imageability and ambiguity have been inconclusive, with one study showing larger imageability effects for unambiguous words (Tokowicz & Kroll, 2007), corresponding to the numerical trend in our error data, while another study showed imageability effects to be stronger for homonymous words (Rubenstein et al., 1970). More generally, our results are consistent with many previous studies that have found imageability effect without consideration of the ambiguity of the stimuli (e.g., Balota, Cortese, Sergent-Marshall, Spieler, & Yap, 2004; Evans et al., 2012; James, 1975). Such effects fit with the idea that highly imageable words have richer semantic representations (Plaut & Shallice, 1993; Wiemer-Hastings & Xu, 2005) and our results indicate that the imageability effect does not arise solely from differences in contextual availability/variability (cf. (Schwanenflugel, Harnishfeger, & Stowe, 1988; Schwanenflugel & Shoben, 1983). Positive imageability effects were also observed in the relatedness decision task but these were weak (significant only by participants in RTs). Given that we controlled for SemD, it is possible that previous observations of imageability effects in semantic tasks could be in part due to the negative effects of high SemD on low imageability words. On the other hand, Hoffman et al. (2013) observed independent effects of both SemD and imageability in a synonym judgment task in which participants selected semantically related words from a choice of three (e.g., is *chance* similar to *logic, theory,* or *risk*?). An alternative possibility, therefore, is that the magnitude of the imageability effect may be task dependent. Imageability effects are typically larger in lexical decision tasks than in reading aloud, for example (Balota et al., 2004; Cortese & Khanna, 2007). Our study is the first to explore the impact of imageability using the relatedness decision task. Direct cross-task comparisons of imageability effects are needed to resolve this issue.

Finally, we note that effects of SemD and imageability have also been found in a subset of neuropsychological patients with semantic impairments and the presence or absence of these effects in different individuals may shed further light on the underlying processes involved. One group of patients who show strong effects of SemD are aphasic individuals who have semantic deficits following stroke (semantic aphasia; Head, 1926). These patients, have difficulty activating the appropriate aspects of their semantic knowledge for the task in hand; for example, when asked to name a picture of a squirrel, they may say “nuts” (Jefferies & Lambon Ralph, 2006). We recently analyzed the factors influencing semantic processing in these patients using the synonym judgment task (Hoffman, Rogers, & Lambon Ralph, 2011). Patients showed robust effects of both imageability and SemD, with SemD being the strongest single predictor of performance. Patients were less likely to respond correctly to high SemD words. Interestingly, semantic deficits in this patient group have been linked with poor executive regulation of semantic knowledge (Jefferies & Lambon Ralph, 2006; Noonan, Jefferies, Corbett, & Lambon Ralph, 2010). This suggests that executive regulation may be particularly important when comprehending high SemD words. This possibility could be explored in future models of semantic ambiguity. For example, top-down executive processes may play a role in cleaning up the noisy semantic patterns elicited by high SemD words or in constraining their patterns of activation according to the current context.

At the same time, it is important to note that poor processing of high SemD words is not a universal consequence of brain damage. Patients with semantic dementia have severe semantic deficits that result from gradual degradation of the semantic knowledge store (Patterson, Nestor, & Rogers, 2007). When we analyzed semantic judgment performance in a group of semantic dementia cases who were matched to the semantic aphasics on overall accuracy, we found no effect of SemD in this group (Hoffman et al., 2011). These patients were instead influenced primarily by the frequency and imageability of the words. The consistent influence of imageability combined with the variable influence of SemD across patient groups indicates that these two dimensions of meaning are independent and that particular difficulty with highly diverse words is not an automatic consequence of semantic impairment. In general, we believe that the ability of the SemD measure to account for performance in normal and disordered language processing demonstrates its value as a psycholinguistic marker of semantic ambiguity.

## Figures and Tables

**Table 1 tbl1:** Average Psycholinguistic Properties of Word Stimuli Used in Experiments 1 and 2 and Nonword Stimuli Used in Experiment 1

Property	HIHD	HILD	LIHD	LILD	Nonwords
Imageability	548 (57)	559 (56)	341 (35)	343 (34)	—
SemD	1.83 (.09)	1.40 (.16)	1.84 (.08)	1.41 (.18)	—
Log frequency	1.06 (.39)	1.01 (.38)	1.04 (.38)	1.03 (.40)	—
Subjective frequency	389 (72)	386 (68)	394 (70)	393 (68)	—
Age of acquisition	401 (51)	408 (49)	408 (51)	413 (57)	—
Orthographic neighbors	6.10 (4.38)	6.15 (5.20)	6.65 (4.73)	6.67 (5.28)	5.40 (4.18)
Length	4.57 (.75)	4.47 (.85)	4.50 (.85)	4.43 (.79)	4.65 (.83)
Bigram frequency	1223 (847)	1215 (962)	1193 (892)	1351 (980)	1381 (1425)
Homonyms	20%	23%	15%	17%	—
*Note.* Standard deviations are shown in parentheses. HI = high imageability; LI = low imageability; HD = high semantic diversity; LD = low semantic diversity.					

**Table 2 tbl2:** Average Psycholinguistic Properties of First Words Used in Experiment 2

Property	HIHD	HILD	LIHD	LILD
Imageability	556 (89)	568 (97)	366 (62)	382 (71)
SemD	1.79 (.18)	1.53 (.25)	1.86 (.21)	1.56 (.23)
Log frequency	1.48 (.56)	1.46 (.57)	1.40 (.63)	1.34 (.63)
Subjective frequency	421 (111)	418 (175)	456 (91)	423 (146)
Age of acquisition	356 (68)	351 (91)	386 (97)	387 (99)
Orthographic neighbors	6.35 (5.86)	6.27 (5.85)	5.43 (5.24)	5.53 (5.22)
Length	4.73 (1.13)	4.67 (1.13)	4.67 (1.19)	4.63 (1.21)
Bigram frequency	1353 (1033)	1689 (1717)	1516 (1997)	1265 (1088)
Target production rate in free association	2.38% (5.36)	1.71% (4.75)	2.11% (5.75)	2.78% (8.71)
*Note*. Standard deviations are shown in parentheses. HI = high imageability; LI = low imageability; HD = high semantic diversity; LD = low semantic diversity.				

## Footnotes

1 In a later experiment in the same article, Pexman et al. compared ambiguous words whose meanings were judged to be more or less related. As this experiment was conducted in Japanese, we have not been able to investigate the SemD of the words used. However, given that the words were selected by the same method of asking participants to judge whether they have more than one meaning, it is likely that these words were also primarily ambiguous in the homonymous sense.

2 A reviewer asked whether we would have obtained similar results if we had used sparser semantic patterns. To investigate this, we ran supplementary simulations using a semantic layer of 200 units in which each prototype consisted of five activated units, related words shared four semantic units and unrelated words shared none. All other simulation parameters were unchanged. This yielded similar results to the main simulation reported here.

## A Experimental Stimuli

**Table:**

Condition	Unrelated first word (Expt 2)	Related first word (Expt 2)	Target (Expts 1 and 2)
High imageability, High SemD	paper	circle	arc
	strike	tag	badge
	money	sit	bench
	picture	ticket	booth
	avoid	support	brace
	circle	money	buck
	camp	food	choke
	mask	stir	churn
	stem	chime	clock
	boot	machine	crane
	camel	stop	curb
	slice	grave	dead
	winter	scratch	dent
	dig	avoid	dodge
	bite	paper	fold
	jewel	picture	frame
	scratch	winter	freeze
	cellar	divide	gap
	pad	jewel	gem
	divide	dig	grave
	food	watch	guard
	ruin	host	guest
	host	swing	hinge
	climb	mask	hood
	support	camel	hump
	machine	hut	lodge
	ticket	cover	mask
	track	grass	moss
	flash	climb	mount
	watch	repair	patch
	shred	top	peak
	fire	spike	pierce
	repair	lump	pile
	cash	board	plank
	grass	cash	purse
	reach	stem	root
	swing	fire	sack
	grave	reach	scope
	silver	camp	scout
	tie	rip	shred
	rip	pad	sketch
	strand	strike	slap
	weave	flash	spark
	tag	boot	spur
	split	post	stamp
	hut	silver	steel
	cover	van	steer
	chime	cellar	store
	collar	shred	strip
	rub	tie	suit
	spike	strand	thread
	stop	bite	tick
	heart	collar	tie
	board	split	torn
	stir	track	trail
	lump	heart	vein
	sit	weave	web
	post	slice	wedge
	van	rub	wipe
	top	ruin	wreck
High imageability, Low SemD	cough	sound	bass
	waist	king	boss
	rain	hill	brow
	ice	bed	bunk
	hill	ride	cab
	group	stomach	chest
	roll	tribe	clan
	bronze	ship	crew
	holy	sleeve	cuff
	hay	roll	dice
	wood	meat	flesh
	heat	bird	flight
	scream	wind	flute
	forest	ice	froze
	tavern	bake	fry
	window	heat	grill
	stitch	ocean	gulf
	kitchen	kill	gun
	slant	waist	hip
	bake	scream	howl
	itch	tavern	inn
	bed	leg	jeans
	coffin	sprint	jog
	sound	dress	lace
	war	race	lap
	dress	window	ledge
	scarf	rain	mist
	make	hole	mole
	sprint	girl	niece
	jacket	vermin	pest
	leg	forest	pine
	gin	soil	plant
	stomach	tremor	quake
	weapon	rod	reel
	meat	fold	ridge
	stare	cook	roast
	tribe	holy	saint
	soil	gin	scotch
	bird	bolt	screw
	ocean	scarf	shawl
	hole	clothes	silk
	vermin	jacket	sleeve
	king	slant	slope
	ink	cough	smoke
	wind	stare	squint
	ship	ink	stain
	army	make	stew
	rod	needle	stitch
	tremor	wood	stump
	girl	bronze	tan
	torso	weapon	tank
	needle	kitchen	tile
	fold	coffin	tomb
	clothes	war	trench
	bolt	group	tribe
	ride	army	troop
	race	torso	waist
	kill	stitch	weave
	sleeve	hay	wheat
	cook	itch	wool
Low imageability, High SemD	hung	set	batch
	vow	void	blank
	stagger	gust	blown
	size	nod	bob
	end	trick	bribe
	wrong	push	budge
	glide	lie	cheat
	depth	treat	cure
	set	swift	dash
	bad	height	depth
	clue	sway	drift
	sturdy	plain	dull
	move	bogus	fake
	blur	hungry	fed
	jump	broke	fix
	mass	fail	flop
	height	current	flow
	plus	move	forth
	push	worry	fuss
	select	joy	glee
	swift	dull	gloom
	trick	end	halt
	hum	heave	haul
	wrath	sick	heal
	creep	weak	mild
	gust	pinch	nip
	excite	select	pick
	current	mercy	plead
	barter	vow	pledge
	worry	ask	pose
	docile	clear	pure
	hungry	fear	scare
	ask	wrath	scorn
	clear	modest	shy
	nod	blur	smear
	leapt	steal	snatch
	modest	creep	sneak
	struggle	sharp	sour
	sway	bad	spoil
	plain	jump	sprang
	broke	leapt	sprung
	straight	wait	stay
	fail	wrong	steal
	joy	straight	stiff
	fear	struggle	strain
	weak	hung	strung
	help	plus	sum
	sharp	depth	sunk
	sick	barter	swap
	dash	stagger	sway
	pinch	dash	swift
	wait	glide	swoop
	treat	docile	tame
	void	excite	thrill
	bogus	mass	ton
	steal	sturdy	tough
	mercy	clue	trace
	lie	hum	tune
	heave	help	warn
	dull	size	weigh
Low imageability, Low SemD	mute	sore	ache
	fibre	hit	bash
	breathe	join	bind
	sigh	praise	bless
	wobble	fibre	bran
	love	cool	chill
	voice	toss	chuck
	ode	feud	clash
	stink	tap	click
	goal	mute	deaf
	buzz	love	dear
	noise	act	deed
	slept	munch	dine
	go	drug	dose
	crime	thirst	drought
	thirst	deaf	dumb
	float	belief	faith
	cold	hover	flew
	join	stupid	fool
	language	ill	frail
	hurt	float	glide
	hit	sorrow	grief
	munch	sigh	groan
	rear	roar	grunt
	shone	rear	hind
	feud	buzz	hum
	prose	shone	lit
	cool	goal	miss
	tune	word	noun
	mile	cold	numb
	hover	contract	oath
	sorrow	census	poll
	sniff	fury	rage
	tax	noise	rang
	divine	ode	rhyme
	smell	trot	rode
	word	smell	rot
	crease	voice	sang
	toss	stench	scent
	pact	tennis	score
	dare	shout	screech
	shout	crease	seam
	census	wobble	shook
	deaf	breathe	sigh
	roar	crime	sin
	act	bang	slam
	belief	sniff	smell
	fury	divine	soul
	tennis	stink	stench
	sore	dare	stunt
	contract	tune	sung
	ill	sin	theft
	stupid	hurt	thump
	sin	go	trot
	bang	pact	truce
	drug	language	verb
	stench	prose	verse
	trot	tax	wage
	tap	mile	width
	praise	slept	woke

## B Nonword Foils in Experiment 1

ald, ane, ast, baip, balt, bamb, bant, beab, binch, bisle, blain, blist, blop, blurf, bome, boop, bork, brack, brance, brapse, brate, brate, braugh, breat, breeg, breet, bress, broop, bule, bursh, cerge, ceste, cet, chale, chamb, chass, chate, chinx, choarse, chon, chot, chrub, cike, claff, claik, clobe, clomp, corg, couge, cound, crail, creesh, creet, crem, cright, crim, croke, crough, crought, doarse, dranp, drawt, dreed, drep, drobe, dure, duve, eaf, fatch, fent, fint, firce, flact, flinp, floof, flox, fraw, frawl, frymn, gat, ghast, glan, gless, glice, glod, glouse, goap, goll, grabe, gralf, gralm, gramp, grank, grat, gree, gresp, groast, grobe, grourge, gruice, gruite, gruy, guartz, guck, hamp, hant, hap, harch, heaf, hidge, hidst, hort, hoy, jate, jung, knige, kross, lail, lirm, lomp, lork, lown, mafe, mant, milt, muth, narve, natch, nate, neize, nog, noss, nuise, oal, panch, pard, pearb, ped, pept, pib, pire, plamb, plape, plast, plose, prabe, preeze, pret, prob, pult, pund, quase, rall, rarch, rart, rea, reb, reeg, reem, reft, rell, rew, rint, runc, screap, scrin, scrup, sein, sheal, sheek, shelt, shet, shick, shoft, shrag, shull, siete, skear, skick, sleb, sleg, sloy, smirt, snait, snat, snelt, spalt, splish, squesh, starge, steat, steck, steeg, steg, stont, stoove, strat, streap, strug, surt, sweeb, swerd, sworp, tain, teaf, tet, thatt, thrave, threl, tibe, toor, torm, tove, tralp, treer, trept, trest, trier, tringe, trit, troap, trompt, trope, tround, troup, trow, trown, trung, tuct, vape, vitz, vooth, wesh, whib, woal, wud, yape, yock, yug, yush.
